# Comprehensive Evaluation and Analysis of Human Settlements’ Suitability in the Yangtze River Delta Based on Multi-Source Data

**DOI:** 10.3390/ijerph20021354

**Published:** 2023-01-11

**Authors:** He Liu, Xueming Li, Yingying Guan, Songbo Li, He Sun

**Affiliations:** 1 School of Geography, Liaoning Normal University, Dalian 116029, China; 2 School of Resource and Environmental Sciences, Quanzhou Normal University, Quanzhou 362000, China; 3 Normal College, Shenyang University, Shenyang 110044, China; 4 National Marine Environmental Monitoring Center, Dalian 116023, China

**Keywords:** human settlements, suitability evaluation, spatial differentiation characteristics, multi-source data, Yangtze River Delta

## Abstract

The suitability of human settlements is critical for quality of life and regional development. As comprehensive evaluations and research on the suitability of human settlements are lacking, a comprehensive evaluation of human settlements in the Yangtze River Delta (YRD) was carried out in 2020 by combining natural and human environmental elements based on multi-source data such as digital elevation models, Landsat remote sensing images, meteorological station data, and points of interest, other multi-source data, and constructions of the human settlements’ suitability indexes. The results showed the following: (1) The spatial suitability of the natural environment in the YRD is significantly affected by the topographic conditions and distance from the sea, showing an increasing spatial differentiation from southwest to northeast, with Shanghai and Yancheng having the best natural environment suitability. (2) The suitability of the human environment in urban areas is better than that in non-urban areas and shows a decreasing trend from the south to the north circle. Shanghai, Zhoushan, and Huaibei have the best human environment suitability. (3) The comprehensive suitability of human settlements includes both the spatial differentiation characteristics of the suitability of natural and human environments. Shanghai and Zhoushan have the mosy comprehensive suitability for human settlements, while Huaibei and Xuzhou have the worst. (4) Land with a comprehensive suitability for human settlements of greater than 0.580 accounts for 23.60% of the total and contains 30.08% of the population and 32.31% of the economy, indicating that areas with a high suitability index have been fully utilized, and the populations and economies with human settlements suitability have a high degree of matching.

## 1. Introduction

The acceleration of globalization, industrialization, and urbanization has led to continuous population expansion. While the widespread population scale brings opportunities for regional development, it also brings challenges, including serious air pollution, intensified heat-island effects, insufficient infrastructure, and urban sprawl [1,2,3]. Thus, the United Nations General Assembly proposed the Sustainable Development Goals (SDGs), aiming to promote the sustainable development of regional social, economic, and environmental components [4]. SDG11 proposes the construction of sustainable cities and communities; the suitability of human settlements, a key indicator when assessing regional sustainability [5], has attracted attention and is important as a reference for the promotion of regional sustainable development [6,7,8].

The human settlement is a surface space that is closely related to people’s daily lives [9]. With the acceleration of urbanization and industrialization, the population has gradually increased, accompanied by an increasing demand for resources and increasing environmental demands, leading to the gradual prominence of the problems of human settlements [10] and impacting on the quality of residents’ lives. Human settlements’ suitability affects the distribution of regional populations [11]. Quantitative research on the suitability of human settlements is of great significance when optimizing the distribution of regional populations, promoting the coordinated development of the region, and building livable and business-friendly cities.

Since the scientific establishment of human settlements, studies related to human settlements have gradually become a hot topic. Extensive research has been carried out on the suitability of human settlements [12,13,14,15], taking mountainous cities [16], watershed cities [17], coastal cities [18] and other areas as research objects, and starting from the perspectives of nature and society [18,19,20]. With the help of an econometric model and geographic information system, a visual analysis was carried out on the suitability of human settlements [21,22] to explore temporal and spatial distribution characteristics [23,24] and predict future development trends [25]. In general, early studies on the suitability of human settlements were mainly based on official statistical data at the macroscale [26,27,28]. With the rise and application of 3S technology, many researchers began to conduct refined studies on the suitability of human settlements based on raster data [21], and their research perspectives mainly focused on an analysis of the suitability of natural environments [29], such as topography, hydrological environment, and vegetation cover [30,31,32]. However, few studies have conducted a comprehensive suitability evaluation of the human environment [33]. The natural environment generally indicates the livability of residents’ living spaces, whereas the human environment reflects the residents’ choices and adaptations to the natural environment. The organic combination of the livability of the natural environment and the suitability of the human environment can more scientifically reflect the actual utilization of human settlements [34]. In summation, the evaluation of the suitability of human settlements mainly focuses on the natural environment elements, while few studies combine the human environment elements, which leads to problems such as an imperfect index system and incomplete evaluation results, and the accuracy of the research is affected to a certain extent. In addition, there are few empirical studies on the suitability of human settlements at the scale of urban agglomeration. Urban agglomeration, as the product of urbanization in its later stage, can exert an important influence on a country’s overall development [35]. Therefore, empirical research on the comprehensive suitability of human settlements at the scale of urban agglomeration has important theoretical and practical significance.

The Yangtze River Delta (YRD), an economic development area on the east coast of China, plays an important role in the sustainable development of China. In addition, as the sixth largest urban agglomeration in the world, its international status and influence are also prominent. In recent years, the population, economy, and land in the YRD have developed rapidly; simultaneously, several problems, such as the deterioration of the ecological environment and the high consumption of energy and resources, have brought challenges to its sustainable development [36]. Thus, a study on the suitability of human settlements in the YRD is of great significance for improving the well-being of residents and enhancing the quality of regional development. It also has a certain reference significance for the construction and development of other urban agglomerations.

Therefore, this study uses the YRD as the research object to combine natural and human environmental elements, and based on multi-source data such as digital elevation models, Landsat remote sensing images, meteorological station data, and points of interest (POI), this study constructs a comprehensive suitability index for human settlements in the YRD from the perspectives of the natural and human environments and quantitatively evaluates the suitability of human settlements in the YRD, which can improve the accuracy and scientific foundation of the study to a certain extent. On this basis, the entropy method and GIS spatial analysis methods were used in a quantitative evaluation of the spatial differentiation law of human settlements’ suitability in the YRD, and its coordination relationship with economic and population distribution was discussed on this basis to rationally guide the population distribution and promote the coordinated development of the regional population and environment, providing a theoretical basis for the optimization of human settlements in the YRD.

## 2. Research Methods and Data Sources

### 2.1. Study Area

**Figure 1:**
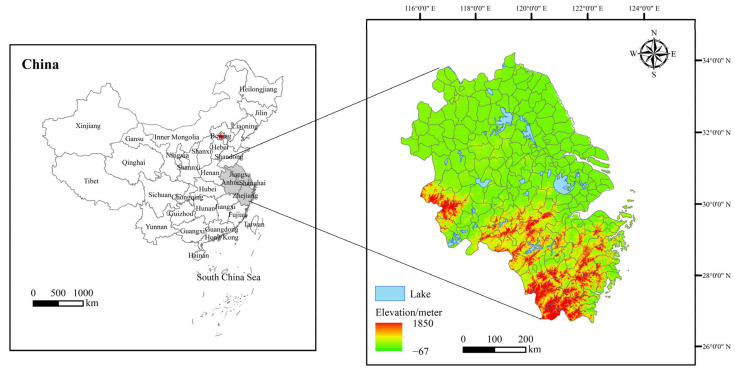
Study area.

The YRD is located in the coastal region of China and is an important intersection of the Belt and Road and the Yangtze River Economic Belt. According to the Outline of the Yangtze River Delta Regional Integration Development Plan (http://www.gov.cn/zhengce/2019-12/01/content5457442.htm, accessed on 15 October 2022), the YRD includes Shanghai, Jiangsu Province, Zhejiang Province, and Anhui Province, with a total of at least 41 prefecture-level cities, covering an area of 358,000 km^2^, and accounting for 3.72% of China’s total area (Figure 1). In 2020, the permanent population of the YRD was 235 million, accounting for 16.1% of the total population of mainland China, and its GNP reached 24,471,318 billion Yuan, accounting for 24% of China’s GDP. It is one of the regions with the most dynamic economic development, the highest degree of openness, and the strongest innovation ability in China [37,38]. The YRD plays a pivotal strategic role in China’s modernization drive and all-around globalization.

### 2.2. Index System Construction and Weight Determination

**Figure 2:**
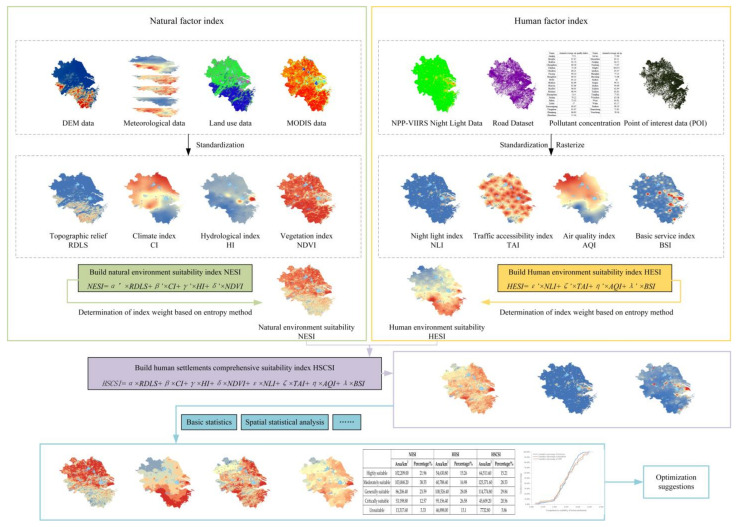
Evaluation framework of HSCSI in the YRD.

As a complex, giant system, regional human settlements include many elements, and, in general, these elements can be divided into two categories: natural and human elements [39]. The natural elements constitute the basis of human settlements, and the human elements are the result of human creation and construction. The two complement each other and constitute human settlements. Based on this, the suitability index system of human settlements is constructed from the perspectives of natural and human environments by referring to relevant studies [11,13,33]. The natural environment includes a topographic relief (RDLS), climate index (CI), hydrological index (HI), and vegetation index (NDVI), whereas the human environment includes a night light index (NLI), traffic accessibility index (TAI), air quality index (AQI), and data basic service index (BSI) (Figure 2).

In terms of index weight determination, the entropy method, as an objective weighting method, can overcome the speculation of subjective weighting and is suitable for the comprehensive evaluation of multiple indicators [40]. Therefore, the entropy method was used to determine the index weight. First, ArcGIS was used to randomly generate 3111 points within the YRD, and then the normalized indicators were extracted from the random points. Then, the entropy method was used to determine the index weights. Finally, according to the obtained weights, the natural environment suitability index (NESI), the human environment suitability index (HESI), and the human settlements comprehensive suitability index (HSCSI) were calculated. The evaluation framework is shown in Figure 2.

### 2.3. Data Source and Processing

The research data mainly include remote sensing image data, meteorological monitoring data, traffic network data, POI data, and social statistics data. A detailed description of the data is given in Table 1. The research mainly uses ArcGIS software to conduct a spatial correction, rasterization of vector data, projection processing, and image cropping to unify the spatial resolution to 1 km.

### 2.4. Index Calculation Method

#### 2.4.1. RDLS

The RDLS is the difference between the highest and lowest elevations in a region [42], which can have a negative impact on the regional population and economic development [43]. Therefore, the RDLS was selected as one of the evaluation indexes of human settlements’ suitability; this was calculated using the ArcGIS window analysis method. The calculation formula is as follows:

(1) $$RDLS=ALT/1000+\left\{\left[max\left(H\right)-min\left(H\right)\right]\times \left[1-P\left(A\right)/A\right]\right\}/500$$

where *ALT* is the average altitude of a raster cell; *max(H)* and *min(H)* are the highest and lowest elevations in the raster cell, respectively; *P(A)* is the flat area in the raster cell; and *A* is the total area of the raster cell.

#### 2.4.2. CI

The CI can reflect the physical comfort degree in a region, and there is a positive correlation between the livability degree of a region and the CI [44]. Based on relevant studies [31], the wind efficiency index (WEI) and temperature and humidity index (THI) were selected to construct the CI; this was calculated by the Kriging interpolation in ArcGIS and the raster calculator. The calculation formula is as follows:

(2) $$WEI=-\left(10\sqrt{v}+10.45-v\right)\times \left(33-t\right)+8.55S$$

(3) $$THI=T-0.55\times \left(1-f\right)\times \left(T-58\right),\;T=1.8t+32$$

where *v* is the average wind speed (m/s), *t* is the monthly average temperature (℃), *S* is the number of sunshine hours (h/d), *T* is the monthly average Fahrenheit temperature (℉), and *ƒ* is the monthly average relative humidity of the air (%). The *WEI* and *THI* were graded according to the grading standard [45], and then the range standardization was used to convert them into a value between 0 and 1, and the average value of the two was the *CI*.

#### 2.4.3. HI

Water resources can support social and economic development in the region [46] and are indispensable natural resources for survival and development [30]. In this study, the HI was constructed by regional precipitation and water-area-specific gravity, which were measured using the Euclidean distance and other functions of ArcGIS. The calculation formula is as follows:

(4) $$HI=\alpha P+\beta W_{a}$$

where *P* is the normalized average annual precipitation, *W_a_* is the normalized water area specific gravity, and *α* and *β* are the weights of *P* and *W_a_*, respectively.

#### 2.4.4. NDVI

Vegetation is the basic component of the terrestrial ecosystem, which can have an important impact on regional ecological environment quality [47,48]. The NDVI was used to reflect the vegetation coverage in the YRD; monthly NDVI data were converted into an annual mean value using the raster calculator tool in ArcGIS. The calculation formula is

(5) $$NDVI=\left(\rho_{NIR}-\rho_{Red}\right)/\left(\rho_{NIR}+\rho_{Red}\right)$$

where *ρ_NIR_* and *ρ_Red_* are the reflectance data of the near-infrared band and red band, respectively.

#### 2.4.5. NLI

As a comprehensive index of human factors, the NLI can reflect the intensity of human activities, the level of urbanization, and the form structure of urban construction land [49,50]. This can better reflect the main characteristics of the human environment [33]. For the NLI, this study used the original nighttime light image data (NPP/VIIRS) downloaded from the official website of the National Oceanic and Intergovernmental Oceanic Administration (NOAA); ArcGIS was used for the spatial correction and mask extraction of the data.

#### 2.4.6. TAI

The regional TAI provides convenient conditions for residents to travel and is an important factor affecting residents’ settlement intentions [51]. This study used the time-cost-weighted distance method to measure the TAI in the YRD, referring to relevant studies [52]. The road was classified, the speed set was accordingly, and the number of minutes required to travel 1 km was set as the time cost [53]. Thus, the distance was measured using a grid calculator, distance analysis, and other functions in ArcGIS. The calculation formula is:

(6) cost=1/v

where *cost* is the time cost and *v* is the speed set by all kinds of roads.

#### 2.4.7. AQI

Air quality not only impacts residents’ health but is also a basic factor affecting their choice of residence [54,55]. Since air pollution is mostly the result of human activities, the AQI belongs to the index of human factors [33]. The AQI used by the Ministry of Environmental Protection of the People’s Republic of China was selected to measure the air quality in the YRD [56], and the Kriging interpolation method of ArcGIS was used for interpolation processing.

#### 2.4.8. BSI

The BSI provides convenience in residents’ daily lives, and the completeness of the infrastructure can have an important impact on the suitability of human settlements [11]. This study uses the kernel density analysis method in ArcGIS to measure the degree of infrastructure completeness in the YRD from the perspectives of medical care, science, education and culture, catering services, and shopping services [57].

### 2.5. Establishment of the Model of HSCSI

Based on the standardized treatment of indicators, the entropy method was used to determine the weight of indicators, and the NESI, HESI, and HSCSI were calculated. The calculation formula is

(7) $$NESI=\alpha^{\prime }\times RDLS^{\prime }+\beta^{\prime }\times CI^{\prime }+\gamma^{\prime }\times HI^{\prime }+\delta^{\prime }\times NDVI^{\prime }$$

(8) $$HESI=\varepsilon^{\prime }\times NLI^{\prime }+\zeta^{\prime }\times TAI^{\prime }+\eta^{\prime }\times AQI^{\prime }+\lambda^{\prime }\times BSI^{\prime }$$

(9) $$HSCSI=\alpha \times RDLS^{\prime }+\beta \times CI^{\prime }+\gamma \times HI^{\prime }+\delta \times NDVI^{\prime }+\varepsilon \times NLI^{\prime }+\zeta \times TAI^{\prime }+\eta \times AQI^{\prime }+\lambda \times BSI^{\prime }$$

where *α’*, *β’*, *γ’*, *δ’*, *ε’*, *ζ’*, *η’*, and *λ’* are the single factor weights of each index. *RDLS’*, *CI’*, *HI’*, *NDVI’*, *NLI’*, *TAI’*, *AQI’*, and *BSI’* are standardized indicators. *α*, *β*, *γ*, *δ*, *ε*, *ζ*, *η*, and *λ* are the comprehensive weights of each index (Table 2).

## 3. Results

### 3.1. Spatial Differentiation Characteristics of HSCSI in YRD

#### 3.1.1. Spatial Differentiation Characteristics of Single Elements of NESI

**Figure 3:**
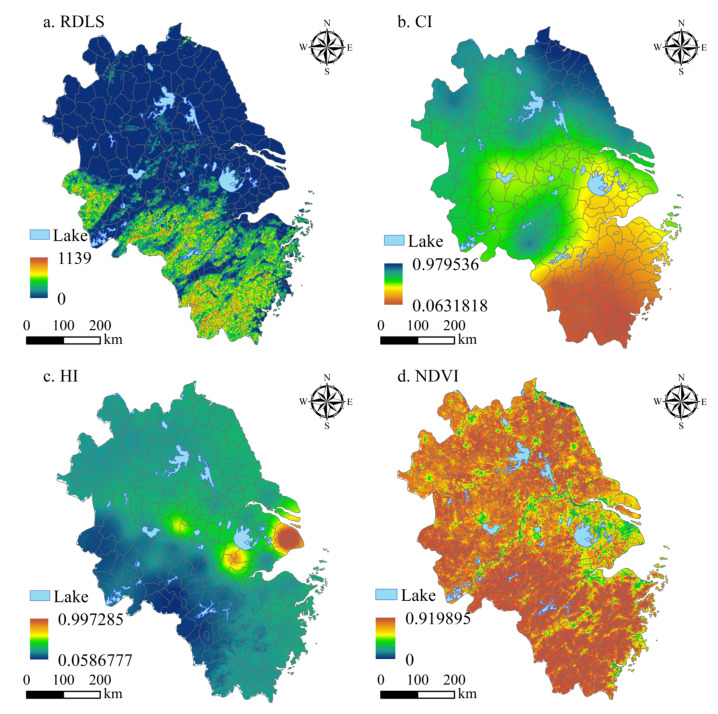
Single-factor spatial distribution of NESI in the YRD.

The spatial distribution of NESI in the YRD is shown in Figure 3. RDLS in the YRD was high in the southwest and low in the northeast (Figure 3a). The southwestern part of the YRD is mostly a mountainous and hilly landform with high relief, while the northeast is mainly the Jianghan Plain and the middle and lower reaches of the Yangtze River Plain with relatively flat topography. In terms of average value, Lishui and Wenzhou have the highest RDLS values, at 391.331 and 298.832 m, respectively. The RDLS values of Nantong and Yancheng are the smallest, at 3.284 and 3.292 m, respectively. The CI shows a gradually increasing trend from south to north (Figure 3b). In terms of average value, the CI values of Lianyungang and Yancheng were the largest, at 0.943 and 0.887, respectively, while those of Wenzhou and Lishui were the smallest, at 0.098 and 0.103, respectively. The HI is the highest in the lower reaches of the Yangtze River, followed by a high-value area near Taihu Lake, and the minimum distribution is in the southwest. A “double core” spatial distribution structure is formed in the space (Figure 3c). The HI of Shanghai is the highest at 0.541, while the HI of Chizhou is the lowest at 0.171. The NDVI is larger in the southwest, followed by the northern region, and is the smallest in the lower reaches of the Yangtze River (Figure 3d). Lishui has the largest NDVI (0.868), and the proportion of urban green space is relatively high. Suzhou (Jiangsu) has the smallest NDVI (0.481), and the green space coverage is relatively low.

#### 3.1.2. Spatial Differentiation Characteristics of Single Elements of HESI

**Figure 4:**
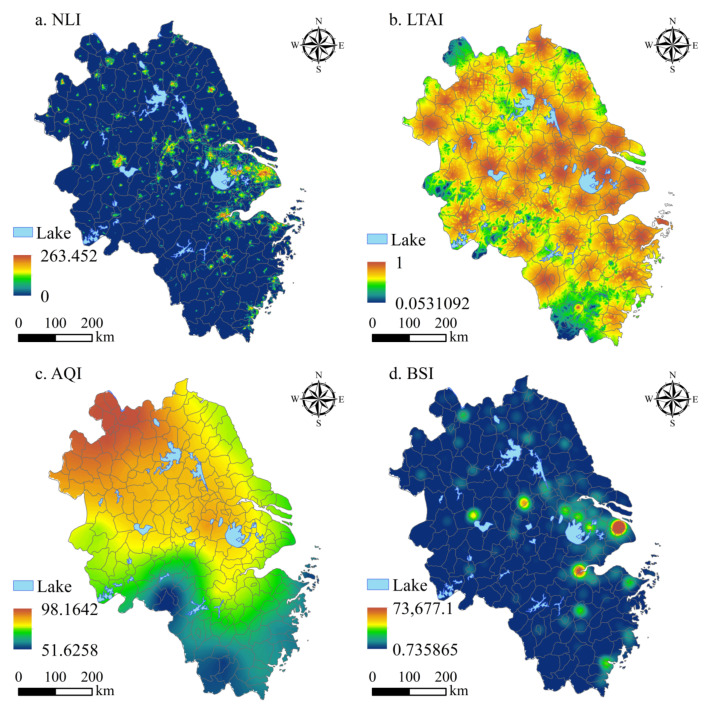
Single-factor spatial distribution of HESI in the YRD.

The spatial distribution of HESI in the YRD is shown in Figure 4. The spatial distribution of NLI in the YRD is substantially different. The NLI in urban areas is significantly higher than that in non-urban areas, showing a multi-core spatial distribution pattern (Figure 4a). The average NLI values of Shanghai and Suzhou (Jiangsu) were the highest, at 17.093 and 11.319, respectively, while the average NLI values of Huangshan and Chizhou were the lowest, at 0.140 and 0.305, respectively. The TAI in the YRD is generally good, showing a decreasing structure from the urban center to the surrounding circles (Figure 4b). The average TAI of Zhoushan is the highest at 0.942, and that of Lishui is the lowest at 0.609. The regional differences in AQI were obvious, gradually increasing from south to north (Figure 4c). Zhoushan, Huangshan, and Lishui in the south had good air quality, while Huaibei, Xuzhou, and Suzhou (Jiangsu) in the north had poor air quality. The BSI has an obvious agglomeration phenomenon, forming a spatial distribution pattern with Shanghai, Wuxi, and Suzhou (Jiangsu) as the agglomeration centers (Figure 4d). Its spatial agglomeration characteristics are similar to NLI, and the BSI in urban areas is significantly better than that in non-urban areas.

#### 3.1.3. Spatial Differentiation Characteristics of NESI, HESI, and HSCSI

**Figure 5:**
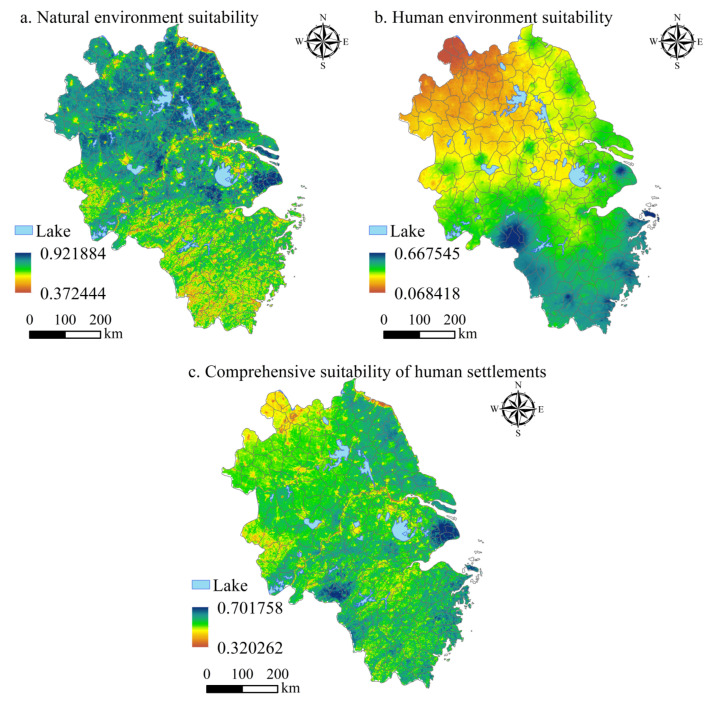
Spatial distribution of natural, human, and comprehensive indexes of human settlements suitability in the YRD.

The suitability of natural, human, and comprehensive settlements in the YRD was quantitatively analyzed based on the entropy method, and a spatial quantitative analysis was carried out by ArcGIS (Figure 5). The spatial distribution of NESI in the YRD shows characteristics of higher distribution in the eastern and northern parts than the western and southern parts, respectively (Figure 5a). Among them, Shanghai, located in the lower reaches of the Yangtze River, and Yancheng, located near the sea, have the best NESI, with the highest value of 0.922, while Lishui and Wenzhou have the worst NESI, with the lowest value of 0.372. The NESI in the YRD is significantly affected by RDLS, CI, and the distance from the sea. The flatter the terrain, the more pleasant the climate, and the closer the distance from the sea, the better the NESI. Therefore, the NESI in the lower reaches of the Yangtze River is better than that in other areas of the YRD.

The spatial distribution characteristics of HESI in the YRD also have the characteristics of NLI, LTAI, AQI, and BSI. Under the comprehensive influence of NLI, LTAI, and BSI, HESI in urban areas is significantly better than that in non-urban areas. In addition, under the effect of AQI, HESI in the YRD showed a decreasing distribution pattern from the south to the north (Figure 5b). The HESI values of Shanghai, Zhoushan, and Huangshan were the best, with the highest value of 0.668, while Xuzhou, Suzhou (Jiangsu), and Huaibei were the worst, with the lowest value of 0.068.

The spatial differentiation characteristics of HSCSI in the YRD are obvious. Its comprehensive natural and human environmental characteristics not only show that the NESI values in the eastern part are higher than those in the western part, but also show that HESI values in urban areas are better than in non-urban areas (Figure 5c). The HSCSI of Shanghai is the highest, with a value of 0.616, while the HSCSI of Xuzhou is the lowest, with a value of 0.494 (Table 3). The lower reaches of the Yangtze River have flat terrain, a low distance from the sea, convenient transportation, good circulation performance of elements, and a high HSCSI. The air pollution in the northern part of the YRD is relatively serious, with low TAI, BSI, and HSCSI.

### 3.2. Spatial Differentiation Characteristics of the Suitability Level of Human Settlements

**Figure 6:**
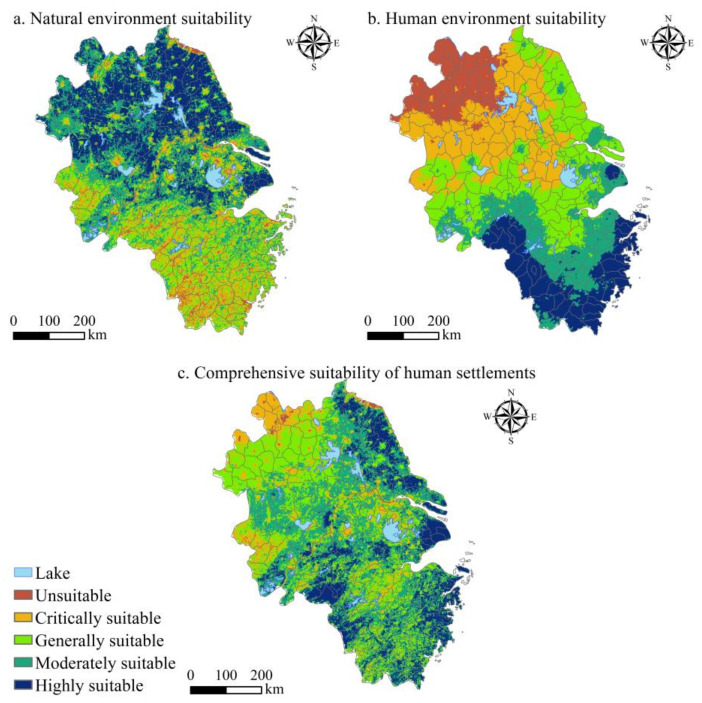
Spatial distribution of natural, human, and comprehensive index levels of human settlements suitability in the YRD.

According to the natural breakpoint method, this study divides the suitability of human settlements in the YRD into five levels (Figure 6, Table 4). The area that is suitable for human settlements in the YRD is about 304,658 km^2^, accounting for 85.10%; the area suitable for the natural environment is about 291,483.60 km^2^, accounting for 81.42% and the area suitable for the human environment is about 215,945.60 km^2^, accounting for 60.32%. This is mainly distributed in the central and coastal areas of the YRD, where the terrain is relatively flat, the AQI is relatively good, and the climate is pleasant. These areas are suitable for residents to live and carry out activities. Therefore, the HSCSI is high. Among them, areas suitable for the natural environment are higher than those suitable for the human environment, mainly due to natural conditions such as RDLS, CI, HI, and NDVI. The critical suitable area is about 45,722.65 km^2^, accounting for 12.74%; the critical suitable area of the natural environment is about 53,198.80 km^2^, accounting for 14.86%; and the critical suitable area of the human environment is about 95,156.40 km^2^, accounting for 26.58%. Mainly distributed in the central and western parts of the YRD, the vegetation coverage is high, the climate is humid, and the air quality is good, all belonging to the critical suitable area. The unsuitable area is about 7732.80 km^2^, accounting for 2.16%; the unsuitable area for the natural environment is about 13.317.60 km^2^, accounting for 3.72%; and the area of the unsuitable area for the human environment is about 47,066.07 km^2^, accounting for 13.10%; this was distributed in the northern part of the YRD, with poor air quality and a low level of transportation convenience, where the human settlements’ suitability is the lowest.

### 3.3. Matching Evaluation of Human Settlements Suitability with Population and Economy in the YRD

Human settlements are closely related to urbanization and economic growth [58]. By referring to relevant studies [13] and starting from the perspective of spatial statistics, the matching degree between the spatial distribution of HSCSI and the spatial distribution of the population and economy can be explored to see whether there is an excessive concentration of population and GDP in the YRD, as well as whether there is room for exploitation in the YRD. Population density and GDP density data were obtained by weighting calculations on the basis of land-use type, night-light brightness, residential density, and population and GDP spatial interaction laws. Compared with the use of only land-use data or night-light data, this can more accurately reflect the distribution information on population and economy [59].

**Figure 7:**
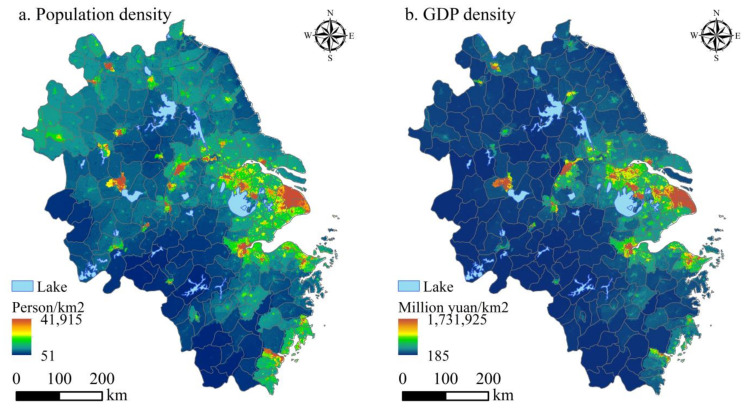
Spatial distribution of population and GDP density in the YRD.

There are similarities between the spatial distribution patterns of population density, GDP density, and HSCSI in the YRD (Figure 7). Generally, urban areas are superior to non-urban areas, and eastern areas are superior to western areas. The spatial correlation coefficients between the HSCSI and population density and GDP density are 0.108 and 0.140 (Table 5). High population and high economic value in the YRD are distributed in the areas with high suitability levels; there is an obvious positive correlation between the HSCSI and population economy. The improvement in HSCSI can effectively promote population agglomeration and economic growth, indicating that the areas with a high level of suitability for human settlements in the YRD have been fully developed.

There is spatial consistency between human settlements’ suitability and population and economy in the YRD (Table 5). As can be seen from Table 5, RDLS, CI, and NDVI are significantly negatively correlated with population and GDP density, while other factors are significantly positively correlated with population and GDP density. The correlation between the BSI and population and economic distribution is the largest, at 0.704 and 0.756, respectively. The correlation between the CI and the population and economic distribution is the smallest, at 0.038 and 0.008, respectively.

**Figure 8:**
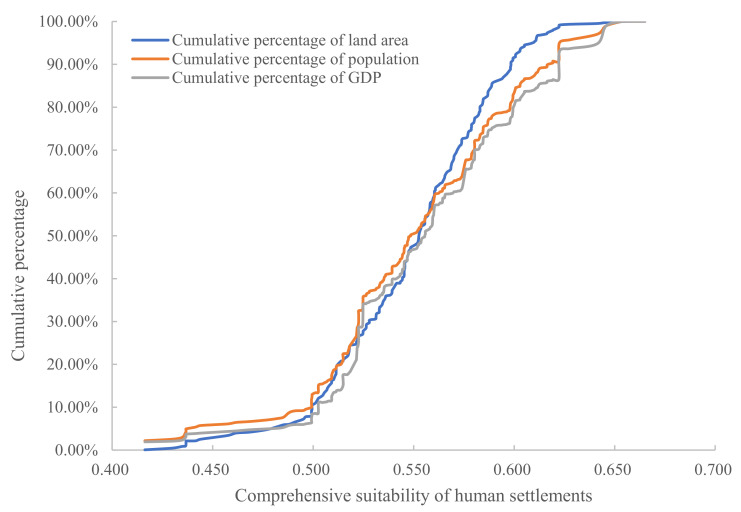
The cumulative percentage of land area, population, and economy in the YRD.

To accurately reflect the consistency and differences between population, economy, and HSCSI in the YRD, this paper takes the HSCSI as the horizontal coordinate and draws the percentage accumulation curve in land area, population, and economy, corresponding to the HSCSI (Figure 8). Among them, the percentage accumulation curves of land area, population, and economy all show an “S” shape structure, and their evolution trends have similar characteristics. All three curves showed a trend from a steep increase to a slow increase, and in the place where the comprehensive suitability index of human settlements was 0.623, the cumulative speed of the three curves slowed down, indicating that the concentration of population and economy in the area with a high comprehensive suitability for human settlements was relatively small; that is, 0.64% of the land held 4.96% of the population and 7% of the economy. Most of the population and economy were concentrated in moderately suitable, generally suitable, and critically suitable areas. However, the three curves do not completely overlap. When the HSCSI is between 0.400 and 0.480, the cumulative percentage of population and economy is higher than that of land area. The 4.76% of the land with a low comprehensive suitability level holds 7.02% of the population and 4.95% of the economy, and the land is in a state of supersaturation. When the HSCSI is between 0.480 and 0.566, the distance between the cumulative percentage of population and economy and the cumulative percentage of the land area is small, and the land is in a state of undersaturation and supersaturation. When the HSCSI is between 0.566 and 0.700, the cumulative percentage of the land area begins to exceed the cumulative percentage of the population and economy, and the land is in a state of undersaturation. In summary, the land with HSCSI values less than 0.580 accounts for 76.40% of the total population and 67.69% of the economy. The land with HSCSI values greater than 0.580 accounts for 23.60% of the total population and 32.31% of the economy. The cumulative percentage difference between land area, population, and economy is not large, indicating that there is a strong degree of matching between the population, economics, and comprehensive suitability distribution of human settlements in the YRD. That is, the HSCSI in the YRD has a significant impact on the spatial distribution of the population and economy.

## 4. Discussion

The Declaration of the United Nations Conference on Human Environment and the SDGs both take the construction of human settlements as an important topic [60], which shows that human settlements have become the focus of global attention [5]. In addition, with the continuous development of globalization and integration, urban agglomeration has gradually become an important carrier of modernization and can have a profound impact on global social and economic development [61]. In this context, it is of great practical significance to explore the construction of human settlements in urban agglomerations. Therefore, this study analyzed the suitability of human settlements in the YRD by integrating multi-source data from the perspectives of nature and humanity, and the research results are significant for the construction of human settlements in the YRD.

Compared with other developed regions in eastern China, the characteristics of human settlements in the YRD are somewhat similar, showing a higher distribution pattern in urban areas than in non-urban areas. Areas with a high suitability for human settlements have been fully utilized, and the distribution of the population and economy and human settlements’ suitability are relatively coordinated [33]. In addition, the HSCSI in the YRD is the result of the joint action of the natural environment and the human environment [2]. Through research, we found that the weaknesses in human settlements’ suitability in the YRD lie in the human environment, and the suitability of the natural environment is better [62]. Therefore, attention should be paid to improving the human environment while constructing human settlements in the YRD. To improve the HSCSI in the YRD, the following suggestions are put forward: (1) strengthen the construction of transport facilities in the southern and northern parts of the YRD and improve transport accessibility in the northern and southern parts; (2) promote the transformation and upgrading of industrial structures by introducing advanced technology and talents, promote the use of clean energy, reduce the use of fossil fuels, and improve regional air quality; and (3) optimize the supply of basic public services in non-urban areas [20], starting from the basic needs of the residents, and promote the rational distribution of basic service facilities. In addition, people living in unsuitable areas should be guided to migrate to more suitable areas [63] to promote the rational distribution of the population and the realization of high-quality economic development; (4) attach importance to the construction of a soft environment for human settlements [11] and improve the human environment without damaging the natural environment to comprehensively improve the overall HSCSI.

At the same time, there are some potential limitations. This study mainly uses objective data to evaluate the suitability of human settlements, but this does not mean that residents are satisfied with the suitability of these settlements [64]. In future research, the residents’ subjective perceptions of human settlements could be explored through questionnaires and other means and combined with the objective environment to explore the suitability of human settlements. Furthermore, human settlements are a dynamic system [10], and exploring their temporal evolution features is vital to improving human settlements. However, this study is only a static study on the suitability of human settlements. In the future, this needs to be extended in the temporal order to improve the literature on human settlements’ suitability.

## 5. Conclusions

In this study, natural and human environmental factors were comprehensively considered, and multi-source data, such as the digital elevation model, Landsat remote sensing image, meteorological station data, and POI, were used to construct an HSCSI. The spatial distribution characteristics of human settlements’ suitability in the YRD in 2020 were analyzed by combining the entropy and GIS spatial analysis methods, and its coordination relationship with the population and economy was explored. The main conclusions are as follows:

(1) The NESI in the YRD is greatly affected by terrain and climate conditions, and the northern region is better than the southern region; HI is best in the lower reaches of the Yangtze River and worst in the southwest. In contrast to HI, NDVI is worst in the lower reaches of the Yangtze River and best in the southwest. In general, NESI in the YRD was significantly affected by RDLS, CI, and the distance from the sea, showing an overall increasing trend from the southwest to the northeast. Shanghai and Yancheng have the best NESI.

(2) The HESI in the YRD was better in urban areas and was greatly affected by the NLI and the degree of infrastructure improvement. TAI is good overall, showing a decreasing trend from the central area of the city to the surrounding layers. AQI showed an increasing trend from south to north. Overall, HESI in the YRD is better than that in non-urban areas and decreases from south to north. Shanghai, Zhoushan, and Huaibei had the best HESI.

(3) The HSCSI in the YRD integrated the characteristics of the natural and human environments. The eastern part of the natural environment is superior to the western part, and the urban part of the human environment is superior to the non-urban part. In summary, HSCSI in Shanghai and Zhoushan was the best, while HSCSI in Huaibei and Xuzhou was the worst.

(4) There is a high degree of match between the HSCSI and the population and economy of the YRD. The spatial correlation coefficients are 0.108 and 0.140, respectively, both at the level of 0.01, and the correlation coefficient between BSI and population and economic distribution is the largest. In summary, land with a comprehensive suitability for human settlements of greater than 0.580 accounts for 23.60% of the total, containing 30.08% of the population and 32.31% of the economy, indicating that land with a high suitability index for human settlements in the YRD was fully utilized and that both population and economy have a high degree of matching in terms of suitability.

## Figures and Tables

**Table 1:** Data sources and description.

Data	Data Type	Year	Data Description	Data Source	Corresponding Index
DEM data	Raster	2020	Digital elevation model with 30 m spatial resolution	http://www.gscloud.cn/, accessed on 10 September 2022.	RDLS
Meteorological station data	Vector	2000–2020	Cumulative annual average of meteorological data such as temperature, relative humidity and precipitation	http://data.cma.cn/, accessed on 7 September 2022.	CI, HI
Land use data	Raster	2020	Remote sensing monitoring decoded data with 30 m spatial resolution [41]	http://doi.org/10.5281/zenodo.4417809, accessed on 1 September 2022.	HI
MODIS data	Raster	2020	Monthly mean data of the normalized vegetation index at 1 km spatial resolution	https://modis.gsfc.nasa.gov/, accessed on 7 September 2022.	NDVI
Nighttime lighting data	Raster	2020	Nighttime lighting data with 500 m spatial resolution (NPPVIIRS)	http://www.noaa.gov/web.html, accessed on 10 September 2022.	NLI
Traffic network data	Vector	2020	For the construction of the TAI	https://www.openstreetmap.org/, accessed on 5 October 2022.	TAI
Environmental monitoring site data	Vector	2014–2020	Annual average air quality index data for 41 prefecture-level cities in the YRD	http://www.cnemc.cn/, accessed on 3 October, 2022.	AQI
POI data	Vector	2020	Including health care, science, education and culture, food services, and shopping services.	https://www.amap.com/, accessed on 9 October 2022.	BSI
Socio-economic data	Vector	2020	Includes statistics on population, economy, land, ect.	China City Statistical Yearbook 2021 (https://navi.cnki.net/knavi/year, accessed on 10 October 2022.)	Population density, GDP density
	Raster	2019	The dataset for population and GDP distribution with 1 km spatial resolution	https://www.resdc.cn/, accessed on 12 October 2022.	

**Table 2:** Weight of each index of HSCSI in the YRD.

System Layer	Indicator Layer	Single Element Weights	Combined Weights
Natural factor index	RDLS	0.387	0.221
	CI	0.024	0.013
	HI	0.361	0.206
	NDVI	0.228	0.130
Human factor index	NLI	0.402	0.173
	TAI	0.200	0.086
	AQI	0.125	0.054
	BSI	0.273	0.117

**Table 3:** Average values of HSCSI in YRD.

City	HSCSI	City	HSCSI	City	HSCSI	City	HSCSI
Shanghai	0.616	Xuancheng	0.558	Huainan	0.541	Changzhou	0.522
Zhoushan	0.614	Ma On Shan	0.556	Chuzhou	0.538	Suqian	0.519
Huangshan	0.586	Lianyungang	0.556	Hefei	0.537	Wuxi	0.517
Taizhou	0.576	Taizhou	0.555	Hangzhou	0.535	Bozhou	0.516
Ningbo	0.574	Introduction	0.554	Huai’an	0.535	Suzhou (Jiangsu)	0.512
Quzhou	0.570	Jinhua	0.551	Nanjing	0.534	Suzhou (Anhui)	0.510
Yancheng	0.569	Wuhu	0.550	Anqing	0.534	Huaibei	0.509
Nantong	0.568	Tongling	0.549	Lu’an	0.529	Xuzhou	0.494
Wenzhou	0.565	Lishui	0.547	Zhenjiang	0.529		
Jiaxing	0.561	Yangzhou	0.547	Bengbu	0.524		
Huzhou	0.560	Chizhou	0.545	Fuyang	0.524		

**Table 4:** Zoning statistics of human settlements suitability in the YRD.

	NESI		HESI		HSCSI	
	Area/km^2^	Percentage/%	Area/km^2^	Percentage/%	Area/km^2^	Percentage/%
Highly suitable	102,209.00	21.96	54,630.80	15.26	64,511.60	15.21
Moderately suitable	103,068.20	38.55	60,788.40	16.98	125,371.60	28.53
Generally suitable	86,206.40	23.59	100,526.40	28.08	114,774.80	29.84
Critically suitable	53,198.80	12.57	95,156.40	26.58	45,609.20	20.56
Unsuitable	13,317.60	3.33	46,898.00	13.10	7732.80	5.86

**Table 5:** Correlation coefficients between human settlement suitability factors and population and GDP density in the YRD.

	Population Density	GDP Density
RDLS	−0.114 **	−0.181 **
CI	−0.038 **	−0.008 **
HI	0.374 **	0.415 **
NDVI	−0.294 **	−0.335 **
NLI	0.513 **	0.587 **
TAI	0.185 **	0.241 **
AQI	0.038 **	0.120 **
BSI	0.704 **	0.756 **
HSCSI	0.108 **	0.140 **

Note: ** indicates significance at the 0.01 level.

## Data Availability

The data presented in this study are available on request from the first author.
